# Effectiveness of a Multifactorial Cardiovascular Risk Reduction Clinic for Diabetes Patients with Depression

**Published:** 2008-09-15

**Authors:** Paul A Pirraglia, Wen-Chih Wu, Lisa B Cohen, Tracey H Taveira

**Affiliations:** Program to Integrate Psychosocial and Health Services in Chronic Diseases and Disability, Providence Veterans Affairs Medical Center; Program to Integrate Psychosocial and Health Services in Chronic Diseases and Disability, Providence Veterans Affairs Medical Center and Warren Alpert Medical School of Brown University, Providence, Rhode Island; Program to Integrate Psychosocial and Health Services in Chronic Diseases and Disability, Providence Veterans Affairs Medical Center and Department of Pharmacy Practice, College of Pharmacy, University of Rhode Island, Kingston, Rhode Island; Program to Integrate Psychosocial and Health Services in Chronic Diseases and Disability, Providence Veterans Affairs Medical Center and Department of Pharmacy Practice, College of Pharmacy, University of Rhode Island, Kingston, Rhode Island

## Abstract

**Introduction:**

Depression may attenuate the effects of diabetes interventions. Our ongoing Cardiovascular Risk Reduction Clinic simultaneously addresses hyperglycemia, hypertension, smoking, and hyperlipidemia. We examined the relationship between depression diagnosis and responsiveness to the Cardiovascular Risk Reduction Clinic.

**Methods:**

We studied Cardiovascular Risk Reduction Clinic participants with diabetes who had a depression diagnosis and those with no mental health diagnosis. Our outcome measure was change in 20-year cardiovascular mortality risk according to the United Kingdom Prospective Diabetes Study (UKPDS) score.

**Results:**

Of 231 participants, 36 (15.6%) had a depression diagnosis. Participants with a depression diagnosis had a higher baseline UKPDS score (56.8 [SD 21.3]) than participants with no mental health diagnosis (49.5 [SD 18.7], *P =* .04). After Cardiovascular Risk Reduction Clinic participation, mean UKPDS scores did not differ significantly (37.8 [SD 15.9] for no mental health diagnosis and 39.4 [SD 18.6] for depression diagnosis). Mean UKPDS score reduction was 11.6 [SD 15.6] for no mental health diagnosis compared with 18.4 [SD 15.9] for depression diagnosis (*P =* .03). Multivariable linear regression that controlled for baseline creatinine, number of Cardiovascular Risk Reduction Clinic visits, sex, and history of congestive heart failure showed significantly greater improvement in UKPDS score among participants with a depression diagnosis (β = 6.0, *P =* .04) and those with more Cardiovascular Risk Reduction Clinic visits (β = 2.1, *P *< .001).

**Conclusion:**

The Cardiovascular Risk Reduction Clinic program reduced cardiovascular disease risk among patients with diabetes and a diagnosis of depression. Further work should examine how depressive symptom burden and treatment modify the effect of this collaborative multifactorial program and should attempt to determine the durability of the effect.

## Introduction

People with depression and diabetes have more cardiovascular disease risk factors than people with diabetes alone (1). The interaction of diabetes and depression is problematic because each negatively influences the other (2). The presence of depression may attenuate the effects of diabetes interventions by decreasing patients' medication adherence and physical activity and by limiting acquisition of diabetes knowledge (3-5). However, depression treatment alone has not been demonstrated to improve diabetes outcomes (4,6-8).

The Cardiovascular Risk Reduction Clinic (CRRC) is an ongoing clinical disease management program at the Providence Veterans Affairs (VA) Medical Center designed for veterans with multiple cardiovascular disease risk factors. We use a multidisciplinary, multifactorial approach to simultaneously address control of hyperglycemia, hypertension, smoking, and hyperlipidemia. The program has been effective for veterans with elevated cardiovascular disease risk factors at the Providence VA Medical Center (9).

We sought to better understand the relationship between responsiveness to the CRRC program and depression diagnosis. Our hypothesis, based on reports in the literature of the impact of depression on diabetes, was that a depression diagnosis would decrease the effectiveness of the program. We used retrospective data from CRRC participants to test this hypothesis.

## Methods

### Study design

We conducted a retrospective cohort study that was approved by the Providence VA Medical Center institutional review board. We obtained data for all CRRC participants enrolled between January 2001 and January 2002 by record extraction and through review of electronic medical records for January 1, 2001, to December 31, 2002. Depression diagnosis (present or not present) was determined by the *International Classification of Diseases, Ninth Revision, Clinical Modification* *(ICD-9-CM)* coding for visits (296.2x, 296.3x, or 311.xx) or mention as a diagnosis in the treating clinician's chart notes. We excluded records that did not have values for body mass index, serum lipids, hypertension, hemoglobin A1c (HbA1c), or smoking status at CRRC baseline. We limited our analysis to patients with diabetes. Because we wished to specifically examine the impact of a depression diagnosis, we limited our scope to patients who had been given a diagnosis of depression but no other associated mental health condition and patients who had no mental health condition at all. Therefore, we excluded patients with a diagnosis of schizophrenia, schizoaffective disorder, bipolar disorder (type 1 or 2), posttraumatic stress disorder, or generalized anxiety disorder, determined from the *ICD-9-CM* coding for visits and from the treating clinician's chart notes.

Demographic data collected were age, sex, and race (coded as white or not). Clinical data included height and weight, active medications, systolic blood pressure (mm Hg), smoking status (current or not), and history or diagnosis of hypertension, stroke, congestive heart failure, chronic obstructive pulmonary disease, or coronary artery disease (prior myocardial infarction, prior admission for unstable angina, coronary artery bypass surgery, or percutaneous coronary angioplasty). Laboratory data collected were HbA1c (%), fasting total cholesterol (mg/dL), fasting high-density lipoprotein cholesterol (HDL, mg/dL), fasting low-density lipoprotein cholesterol (LDL, mg/dL), and serum creatinine (mg/dL).

We determined antidepressant use during CRRC enrollment by prescription of any of the following medications: sertraline, paroxetine, citalopram, fluoxetine, nefazadone, venlafaxine, mirtazapine, or amoxapine. The total number of CRRC visits was also obtained.

To avoid multiple comparisons, we sought to use a single, comprehensive outcome measure applicable to patients with diabetes for cardiovascular disease risk as a metric for CRRC effectiveness. We chose to apply the UK Prospective Diabetes Study (UKPDS) risk engine (10) for 20-year risk of cardiac events as that metric. Therefore, our outcome measure, the effectiveness of the CRRC, was the difference between the baseline UKPDS score and the follow-up score.

The elements that go into the UKPDS risk engine are age at diagnosis of diabetes, sex, "Afro-Caribbean" race, current smoking status, HbA1c, systolic blood pressure, and ratio of total cholesterol to HDL. With the exception of sex, race, and age at diagnosis of diabetes, all data were obtained at baseline and at the time closest to the CRRC discharge date or December 31, 2002, whichever came first. The medical record for the period reviewed did not provide information on when diabetes was diagnosed, so we assumed an onset age of 55 years for all patients, which nullified the age term in the risk calculation.

### The Cardiovascular Risk Reduction Clinic

The CRRC was designed to achieve American Diabetes Association (ADA) guideline-recommended goals for glycemic control, blood pressure control, lipid control, and smoking cessation using education, behavioral counseling, and frequent medication titrations. Clinical pharmacists under the supervision of a cardiologist deliver the program. The CRRC behavioral interventions are based on the Health Belief Model for general behavioral modification counseling (11), the Fagerstrom Tolerance Questionnaire (12), and the Transtheoretical Model of readiness to change for smoking cessation (13). VA clinical pharmacists have prescriptive authority to modify medications under their scope of practice, and they apply previously formulated medication titration algorithms for cholesterol, blood pressure, smoking, and glycemic control to aggressively titrate the medication regimen. A cardiologist is available for immediate consultation for difficult management decisions but does not directly provide care to patients enrolled in the clinic.

At the initial 30-minute visit, CRRC pharmacists assess medication adherence and laboratory parameters and develop a treatment plan to control hypertension, cholesterol, and diabetes. Options for smoking cessation are discussed when applicable. Individualized diet and exercise programs are also created, and referral to a nutritionist and physical therapist is made if necessary. Follow-up sessions of 30 minutes are scheduled every 6 to 8 weeks to monitor adherence and therapeutic effects, reinforce lifestyle modifications, and adjust medications. Diabetic patients are discharged from CRRC once therapeutic goals of systolic blood pressure less than 130 mm Hg, HbA1c less than 7%, total cholesterol less than 200 mg/dL, and LDL cholesterol less than 100 mg/dL are met or nearly met, as recommended by the American College of Cardiology/American Heart Association and ADA guidelines (14-16).

### Statistical analysis

For comparisons between the group with no mental health diagnosis and the group with depression diagnosis, we used χ^2^ tests for categorical variables, *t* tests for normally distributed continuous variables, and Wilcoxon rank-sum tests for non-normally distributed continuous variables. The small size of our sample constrained the number of variables we could include in the final multivariable regression model, so we used Mallows Cp method (17) to select the best model in terms of number of covariates and fit.

## Results

Of 375 CRRC participants, 231 had either no mental health condition or a depression diagnosis as the sole mental health condition and were therefore included in this study. Comparison of the 2 groups (Tables 1 and 2) showed that patients with a depression diagnosis were significantly younger, more obese, more likely to be white, and more likely to be taking antidepressant medication. The difference in median number of CRRC visits was not statistically significant: 4 (interquartile range 2-6) for patients with a depression diagnosis and 3.5 (interquartile range 2-5) for patients without a mental health condition. The median time of CRRC enrollment in days also did not differ significantly: 154 (interquartile range 71-310) for those with a depression diagnosis and 185 (interquartile range 72-333) for those without a depression diagnosis. No deaths occurred in either group during the period of observation. Patients with a depression diagnosis had a higher baseline UKPDS score than did patients without a mental health condition (*P* = .047), suggesting a greater cardiovascular disease risk.

At the end of the study period, we found no significant difference between the mean UKPDS score for the group with no mental health diagnosis (37.8 [SD 15.9]) and the group with depression diagnosis (39.4 [SD 18.6]). The mean change in UKPDS score was 11.6 (SD 15.6) for patients with no mental health diagnosis and 18.4 (SD 15.9) for patients with a depression diagnosis (*P* = .03). Baseline UKPDS scores were higher for patients with depression but discharge scores were similar for both groups.

Using Mallows methods for our model selection, we found that the lowest Cp value was for a 5-variable model: depression diagnosis, baseline creatinine, number of CRRC visits, sex, and history of congestive heart failure. The Cp for this model was 0.41, and the *r^2^* value for the model was 0.17. In this adjusted model, having a depression diagnosis was significantly associated with a greater improvement in UKPDS score (β = 6.0, *P* = .04) and greater UKPDS score improvement was associated with a higher number of CRRC visits (β = 2.1, *P *< .001). Addition of age to the adjusted model did not markedly change the point estimate or *P* value for depression diagnosis.

## Discussion

We found that patients significantly improved after participation in the CRRC whether or not they have a diagnosis of depression. Our findings demonstrate that the conventional wisdom regarding the overlap of diabetes and depression is accurate and, more importantly, modifiable. Patients with a depression diagnosis had significantly higher cardiovascular disease risk as measured by a UKPDS score than did patients with no mental health condition, but because they showed greater improvement after participation in our CRRC, their cardiovascular disease risk at discharge was nearly the same as that of patients without a mental health condition. We did not observe a difference in number of CRRC visits or duration of CRRC enrollment between patients with a depression diagnosis and with no mental health diagnosis, suggesting that participants with a depression diagnosis do not drop out preferentially and do not take longer to achieve CRRC goals. These findings demonstrate that our CRRC program is effective in reducing cardiovascular disease risk in patients with diabetes regardless of depression diagnosis.

Depression can be underdiagnosed in primary care (18). The VA system addresses this problem by conducting an annual 2-question screening for depression (19), increasing the likelihood of detection. If our focus were on whether a depression symptom burden or whether meeting criteria for a depression diagnosis affected performance in the CRRC program, underdiagnosis of depression would be a concern. However, we were interested in whether carrying a depression diagnosis (ie, having been labeled as having depression) made a difference in improvement in cardiovascular disease risk reduction because we believed that providers and patients might be influenced by the presence of a depression diagnosis in their management of cardiovascular disease risks. Our findings suggest that this may be the case; patients with a depression diagnosis had significantly worse baseline cardiovascular disease risk but were able to attain a level of risk similar to that of patients without a depression diagnosis on completion of the CRRC.

We believe that having a depression diagnosis did not attenuate the effectiveness of the CRRC because the CRRC design is supportive. It involves frequent contact and follow-up, counseling about cardiovascular health, and responsiveness to particular patient needs related to cardiovascular disease risk. This personal attention may counter the negative effects of having a depression diagnosis. Another potential benefit is that the CRRC team is focused on cardiovascular disease risk reduction and not on competing concerns, which may reduce the stigma that may be associated with a depression diagnosis. Lastly, activities that are promoted within the program — education, healthy diet, and physical activity — may benefit both depression, if still present, and cardiovascular disease risk. However, all of these explanations are speculative, and we do not have supporting data.

Our work has limitations that must be acknowledged. Because this was an observational study with a limited size, all confounding factors may not have been accounted for or included in our model. We relied on a diagnosis of depression in the medical record, which may have resulted in a misclassification bias, though this would have also biased our findings toward the null. We did not have a measure of depression severity, so we cannot determine whether the diagnosed depression was active or in remission. We do not know whether depressive symptom burden or antidepressant medication use changed over the course of participation in the CRRC nor how this would have affected our findings. Although we hoped to also examine the impact of depression treatment, most patients with a depression diagnosis were receiving antidepressants, which also raises the question of whether we are seeing the effect of depression treatment within this group. One might take the magnitude of improvement of patients with depression compared to that of patients without depression as regression to the mean (ie, less improvement was seen in those without depression because they had less room for improvement). However, it is not the magnitude of improvement that is the point of interest but that those with a depression diagnosis had greater risk before the CRRC and improved to a level similar to that of patients without a depression diagnosis on completion of the CRRC program. We must acknowledge that the utility of the UKPDS risk engine for change over time has not been validated. However, individual outcome measures proven to predict cardiovascular disease risk were all improved after the intervention, so the global score was a good summary of the overall improvement in cardiovascular disease risk. Lastly, our population was predominantly male veterans in a closed health care system, which limits generalizability.

Our findings demonstrate that a multifactorial cardiovascular disease risk reduction clinic run by clinical pharmacists was effective in veterans with diabetes and a depression diagnosis. Furthermore, having a depression diagnosis was associated with greater cardiovascular disease risk before participation in the clinic. Our work indicates that having a depression diagnosis should not be considered a barrier to referral or participation in such a program and, in fact, may be a means of identifying other high-risk patients. Further research is necessary to understand the mediating effects of depressive symptom burden and of depression treatment on the effectiveness of multifactorial cardiovascular disease risk reduction programs and on the durability of the effect we observed.

## Figures and Tables

**Table 1:** Baseline Population Characteristics by Depression Diagnosis, Cardiovascular Risk Reduction Clinic Participants, 2001-2002

Characteristic	No Mental Health Diagnosis N = 195	Depression Diagnosis N = 36	*P* value
Mean age, y (SD)	68.8 (9.5)	62.9 (11.2)	.001^a^
Female, %	1.5	2.8	.60^b^
White, %	39.0	58.3	.03^b^
Tobacco use, %	23.6	27.8	.59^b^
BMI, kg/m^2^ (SD)	30.3 (5.0)	33.0 (8.7)	.009^a^
Creatinine, mg/dL (SD)	1.4 (0.8)	1.6 (1.4)	.39^a^
On antidepressant medication, %	15.4	72.2	<.001^b^
Coronary artery disease, %	43.6	41.7	.83^b^
Stroke, %	6.7	19.4	.01^b^
Congestive heart failure, %	21.5	16.7	.52^b^
COPD, %	18.0	8.3	.15^b^

Abbreviations: SD, standard deviation; BMI, body mass index; COPD, chronic obstructive pulmonary disease.

a *P* values are for the *t *test.

b *P* values are for the *χ* ^2^ test.

**Table 2:** Cardiovascular Disease Risk Factors at CRRC Baseline and After CRRC Participation by Depression Diagnosis, 2001-2002

Cardiovascular Risk	No Mental Health Diagnosis			Depression Diagnosis		

	Baseline **N = 188**	Follow-Up N = 180	Change^a^ ** N = 176**	Baseline **N = 39**	Follow-Up **N = 29**	Change^a^ ** N = 28**
HbA1c, % (SD)	8.4 (2.0)	7.4 (1.4)	1.1 (1.8)	8.9 (2.2)	8.0 (2.3)	1.1 (2.0)
LDL, mg/dL (SD)	106.0 (33.0)	93.9 (28.8)	11.6 (31.9)	114.7 (31.3)	91.8 (37.6)	21.8 (40.8)
Total cholesterol, mg/dL (SD)	184.5 (43.5)	167.2 (39.3)	18.2 (41.8)	195.9 (38.5)	172.5 (43.7)	28.5 (48.9)
Systolic blood pressure, mm Hg (SD)	135.0 (16.7)	128.9 (12.8)	6.1 (16.6)	136.6 (14.7)	123.6 (12.3)	12.6 (13.4)
UKPDS score, (SD)^b^	49.5 (18.7)	37.8 (15.9)	11.6 (15.6)	56.8 (21.3)	39.4 (18.6)	18.4 (15.9)
HDL, mg/dL (SD)	39.4 (11.1)	39.1 (10.9)	0.5 (6.1)	37.9 (8.4)	41.2 (11.1)	-2.6 (12.8)
Body mass index, kg/m^2^ (SD)	30.3 (5.0)	30.3 (5.0)	-0.06 (1.4)	33.0 (8.7)	33.2 (9.1)	-0.3 (1.6)
Tobacco use, %	23.6	14.9	8.7	27.8	25	2.8

Abbreviations: CRRC, Cardiovascular Risk Reduction Clinic; HbA1c, hemoglobin A1c; LDL, low-density lipoprotein cholesterol; UKPDS, United Kingdom Prospective Diabetes Study; HDL, high-density lipoprotein cholesterol.

a Change scores are presented only for patients who had both baseline and follow-up scores. Numbers do not total 231 because not all patients had a UKPDS score.

b UKPDS score rates the probability of 20-year cardiovascular mortality.

## References

[1] Katon WJ, Lin EH, Russo J, Von Korff M, Ciechanowski P, Simon G (2004). Cardiac risk factors in patients with diabetes mellitus and major depression. J Gen Intern Med.

[2] McKellar JD, Humphreys K, Piette JD (2004). Depression increases diabetes symptoms by complicating patients' self-care adherence. Diabetes Educ.

[3] Kalsekar ID, Madhavan SS, Amonkar MM, Makela EH, Scott VG, Douglas SM (2006). Depression in patients with type 2 diabetes: impact on adherence to oral hypoglycemic agents. Ann Pharmacother.

[4] Ciechanowski PS, Katon WJ, Russo JE, Hirsch IB (2003). The relationship of depressive symptoms to symptom reporting, self-care and glucose control in diabetes. Gen Hosp Psychiatry.

[5] Murata GH, Shah JH, Adam KD, Wendel CS, Bokhari SU, Solvas PA (2003). Factors affecting diabetes knowledge in Type 2 diabetic veterans. Diabetologia.

[6] Fenton JJ, Von Korff M, Lin EH, Ciechanowski P, Young BA (2006). Quality of preventive care for diabetes: effects of visit frequency and competing demands. Ann Fam Med.

[7] Katon W, von Korff M, Ciechanowski P, Russo J, Lin E, Simon G (2004). Behavioral and clinical factors associated with depression among individuals with diabetes. Diabetes Care.

[8] Lin EH, Katon W, Rutter C, Simon GE, Ludman EJ, von Korff M (2006). Effects of enhanced depression treatment on diabetes self-care. Ann Fam Med.

[9] Taveira TH, Wu WC, Martin OJ, Schleinitz MD, Friedmann P, Sharma SC (2006). Pharmacist-led cardiac risk reduction model. Prev Cardiol.

[10] Stevens RJ, Kothari V, Adler AI, Stratton IM (2001). The UKPDS risk engine: a model for the risk of coronary heart disease in Type II diabetes (UKPDS 56). Clin Sci (Lond).

[11] (2005). National Cancer Institute. Theory at a glance. In: U.S. Department of Health and Human Services. A guide for health promotion practice.

[12] Fagerstrom KO (1978). Measuring degree of physical dependence to tobacco smoking with reference to individualization of treatment. Addict Behav.

[13] DiClemente CC, Prochaska JO, Fairhurst SK, Velicer WF, Velasquez MM, Rossi JS (1991). The process of smoking cessation: an analysis of precontemplation, contemplation, and preparation stages of change. J Consult Clin Psychol.

[14] (2006). American Diabetes Association. Standards of medical care in diabetes—2006. Diabetes Care.

[15] Chobanian AV, Bakris GL, Black HR, Cushman WC, Green LA, Izzo JL (2003). Seventh report of the Joint National Committee on Prevention, Detection, Evaluation, and Treatment of High Blood Pressure. Hypertension.

[16] Gibbons RJ, Balady GJ, Beasley JW, Bricker JT, Duvernoy WF, Froelicher VF (1997). ACC/AHA Guidelines for Exercise Testing. A report of the American College of Cardiology/American Heart Association Task Force on Practice Guidelines (Committee on Exercise Testing). J Am Coll Cardiol.

[17] Mallows CL (1973). Some comments on CP. Technometrics.

[18] Goldman LS, Nielsen NH, Champion HC (1999). Awareness, diagnosis, and treatment of depression. J Gen Intern Med.

[19] Whooley MA, Avins AL, Miranda J, Browner WS (1997). Case-finding instruments for depression: two questions are as good as many. J Gen Intern Med.

